# Electrophysiological Mechanisms and Therapeutic Potential of Calcium Channels in Atrial Fibrillation

**DOI:** 10.31083/RCM33507

**Published:** 2025-06-25

**Authors:** Zuyuan Huang, Cheng Luo, Zimin Wu, Baoshi Zheng

**Affiliations:** ^1^Department of Cardiovascular Surgery, The First Affiliated Hospital of Guangxi Medical University, 530021 Nanning, Guangxi, China

**Keywords:** atrial fibrillation, calcium channels, calcium homeostasis, electrophysiology, therapeutic targets

## Abstract

Atrial fibrillation (AF) is a prevalent and complex arrhythmia for which the pathogenesis involves various electrophysiological factors, notably the regulation of calcium channels. This article aimed to investigate the specific roles and molecular mechanisms of the L-type and T-type calcium channels, ryanodine receptors (RyRs), inositol 1,4,5-triphosphate receptors (IP3Rs), calcium release-activated calcium (CRAC) channels, and transient receptor potential (TRP) channels in the pathogenesis and persistence of AF. In addition, this article reviews recent advances in calcium channel-targeted drugs from experimental and clinical studies, offering new insights into the relationship between calcium channel regulation and AF pathology. These findings suggest promising directions for further research into the mechanisms of AF and the development of targeted therapeutic strategies.

## 1. Introduction

**Fig. 1.**
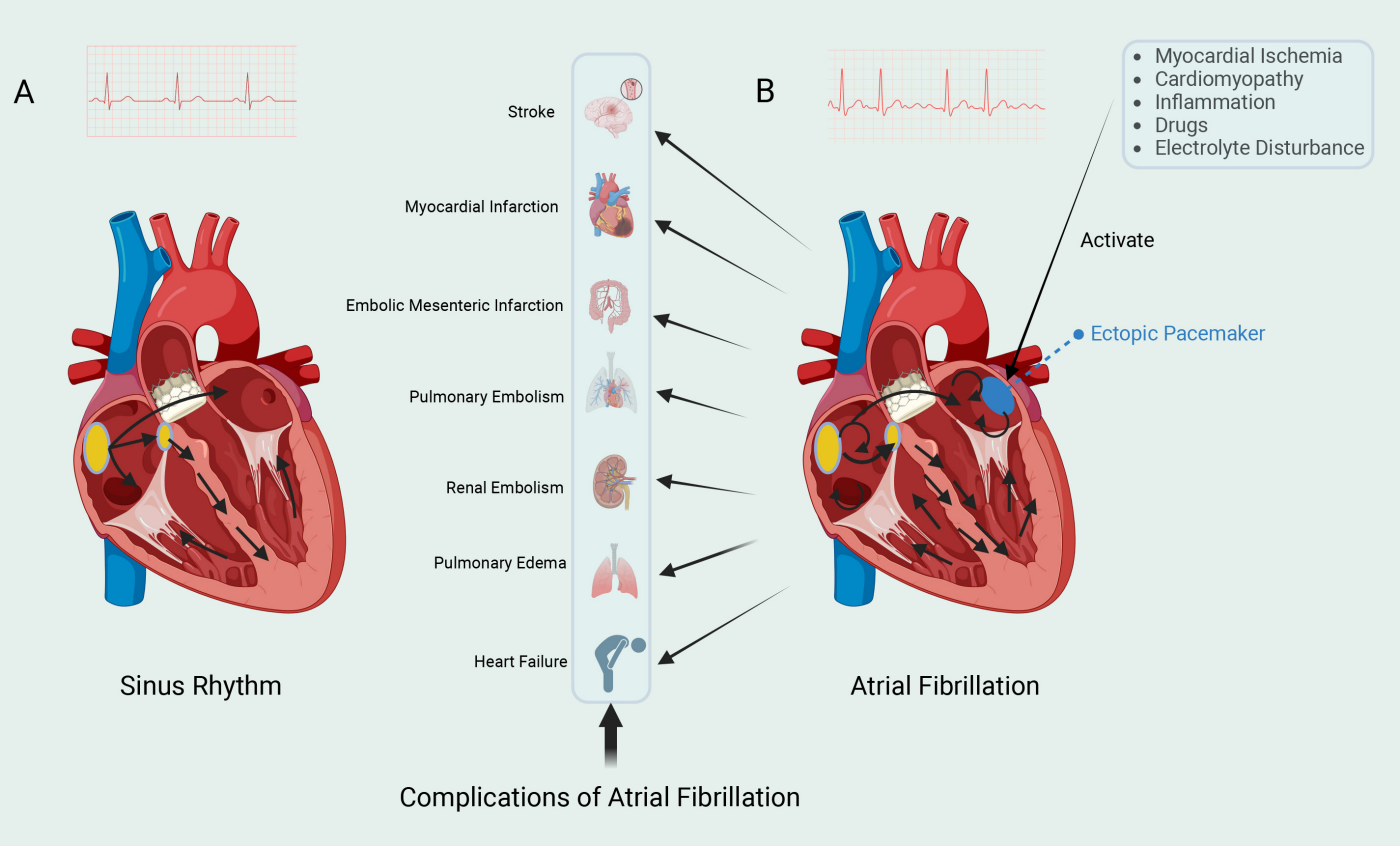
**Two cardiac rhythms**. (A) The left electrocardiogram shows sinus rhythm. The sinoatrial (SA) node, located in the right atrium, can spontaneously generate action potentials. The electrical impulses generated by the SA node are rapidly propagated to both the left and right atria, and then transmitted to the ventricles via the atrioventricular (AV) node. The impulses then spread rapidly throughout the ventricles via the Purkinje fiber system, leading to synchronized contraction of the ventricular muscle and the expulsion of blood. (B) The right electrocardiogram shows atrial fibrillation (AF). AF is typically triggered by spontaneous firing from ectopic pacemaker sites, with the most common trigger originating from the pulmonary veins. These ectopic pacemaker sites are influenced by various factors, which promote the initiation and maintenance of AF. Electrical impulses continuously circulate within the atria, resulting in rapid and irregular electrical activity. AF can lead to various complications. Fig. 1 was drawn using BioRender.

Atrial fibrillation (AF) is one of the most prevalent arrhythmias globally, affecting over 60 million individuals [1]. AF markedly elevates the risk of stroke, heart failure, and various other cardiovascular complications, imposing a substantial burden on healthcare systems and generating significant socio-economic costs [2, 3] (Fig. 1). Despite the availability of multiple clinical treatment strategies, the underlying pathogenesis of AF remains complex and not fully understood. In particular, the regulatory role of calcium channels in AF pathophysiology has received increasing attention.

In the pathophysiology of AF, calcium channels play a crucial role in maintaining calcium homeostasis. There are several types of calcium channels. This article specifically addresses L-type calcium channels (LTCCs), T-type calcium channels (T-channels), ryanodine receptors (RyRs), inositol 1,4,5-triphosphate receptors (IP3Rs), calcium release activated calcium (CRAC) channels, and transient receptor potential (TRP) channels, all of which are closely associated with cardiac function [4, 5, 6, 7, 8, 9]. The regulation of calcium ion balance depends on the interaction and functional maintenance of these calcium channels; dysfunction in these channels is widely regarded as a critical mechanism underlying the pathogenesis of AF [10]. During AF, abnormal expression and function of calcium channels in atrial myocytes lead to intracellular calcium imbalance, further contributing to electrophysiological disturbances and structural remodeling in the atria [11, 12, 13, 14]. Research has demonstrated that dysregulation in calcium channels are closely associated with atrial fibrosis, inflammatory responses, and electrophysiological instability, which collectively promote the initiation and persistence of AF [15, 16].

Calcium channel blockers are widely used in the management of AF, as they help mitigate electrophysiological dysregulation and reduce AF frequency by lowering intracellular calcium concentrations in atrial myocytes [17]. In recent years, novel therapeutic strategies targeting calcium channels, including calcium channel inhibitors and agents that modulate calcium signaling pathways, have demonstrated promising potential [18, 19, 20]. Consequently, in-depth exploration of these mechanisms and the development of targeted treatment strategies are crucial to improving the prognosis for AF patients.

## 2. The Role of Calcium Channels in Cardiac Electrophysiological Activity

AF is a persistent or intermittent arrhythmia resulting from abnormal electrical activity within the atria [21]. Calcium homeostasis, the regulation of intracellular and extracellular calcium concentrations, is crucial for cardiac function and cellular signal transduction [22]. Calcium channels play an essential role in maintaining Ca^2+^ levels. T-channels open during the early depolarization and repolarization phases of cardiomyocytes, allowing a relatively small influx of calcium [23]. Upon depolarization of the cardiomyocyte membrane, LTCCs facilitate the entry of Ca^2+^ into the cell [24]. This increase in intracellular Ca^2+^ concentration activates RyR2 on the sarcoplasmic reticulum (SR), releasing stored Ca^2+^ and triggering calcium-induced calcium release (CICR). This process drives myocardial contraction, ensuring an adequate cardiac output to prevent ischemic injury [25].

Additionally, IP3 binds to IP3Rs in the SR, further increasing intracellular Ca^2+^ levels [26]. To maintain calcium homeostasis, the sarcoplasmic/endoplasmic reticulum Ca^2+^-ATPase (SERCA) actively transports excess intracellular calcium back into the SR, thereby reducing cytosolic calcium levels [27]. Simultaneously, the sodium-calcium exchanger (NCX) contributes to calcium balance by exchanging intracellular Ca^2+^ for extracellular Na⁺, thus regulating calcium concentrations [28]. When intracellular calcium levels decrease, CRAC channels facilitate the entry of extracellular calcium to replenish the intracellular calcium deficit [29]. Therefore, these calcium transport mechanisms ensure normal excitation-contraction coupling and maintain cardiac rhythm. In view of the central role of calcium influx in cardiac electrophysiology, it is necessary to further explore the specific functions of various calcium channels.

### 2.1 LTCCs

**Fig. 2.**
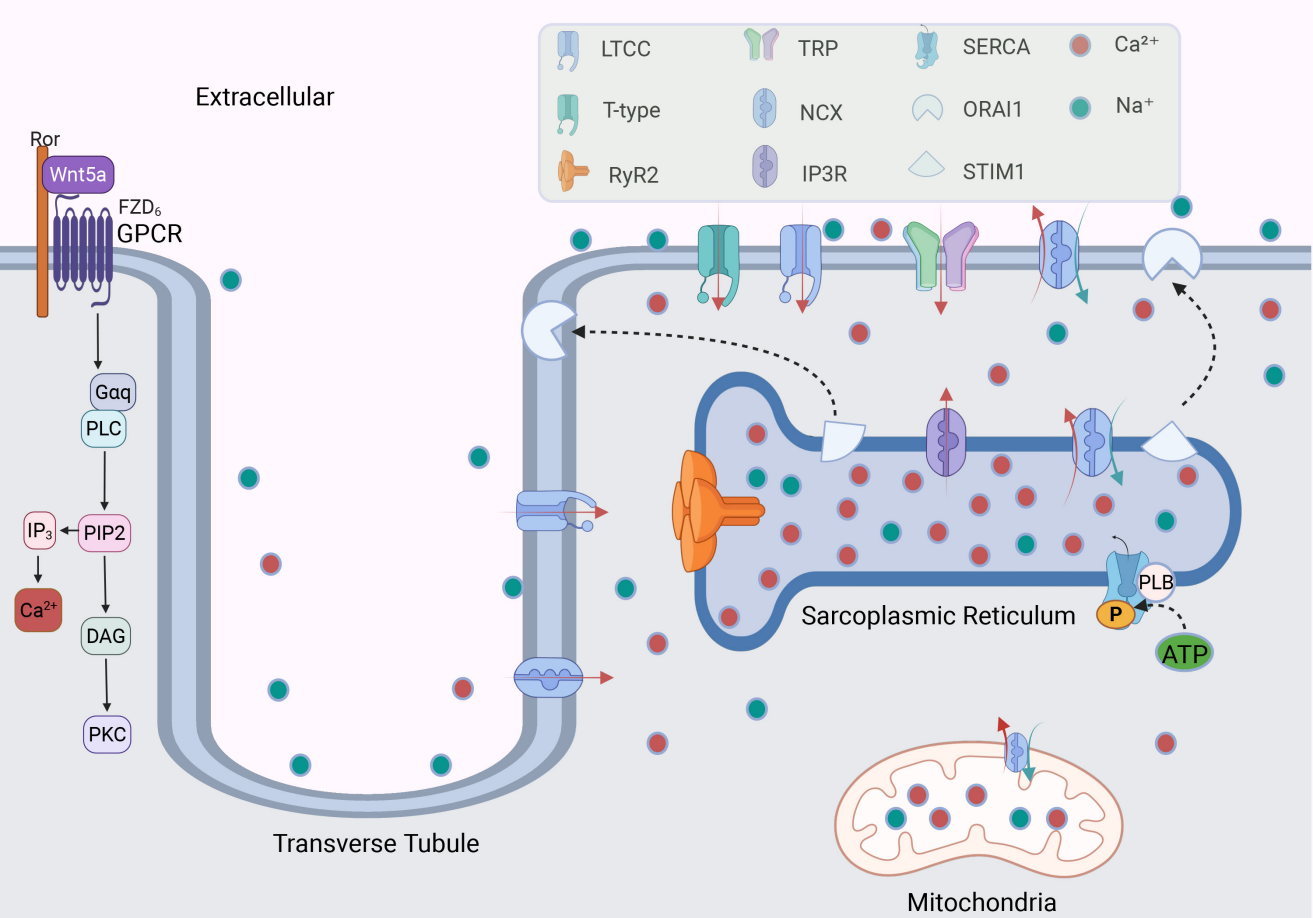
**The mechanism of calcium channels regulating Ca^2+^ in cardiomyocytes**. GPCR regulates the level of intracellular and extracellular Ca^2+^ by activating G protein; calcium channels distributed in cell membranes and organelles regulate the balance of Ca^2+^. The binding of Stromal Interaction Molecule 1 (STIM1) on the sarcoplasmic reticulum (SR) with Orai Calcium Release-Activated Calcium Modulator 1 (ORAI1) on the cell membrane participates in the regulation of calcium ions. The red and green arrows indicate the directions of Ca^2+^ and Na⁺ movement, respectively. LTCC, L-type calcium channel; RyR2, ryanodine receptor 2; TRP channel, transient receptor potential channel; NCX, sodium-calcium exchanger; IP3R, inositol 1,4,5-triphosphate receptor; SERCA, sarcoplasmic/endoplasmic reticulum Ca^2+^-ATPase; PLB, phospholamban; ATP, adenosine triphosphate; P, phosphate; GPCR, G-Protein coupled receptor; Ror, receptor tyrosine kinase-like orphan receptor; Wnt5a, wnt family member 5A; FZD6, frizzled class receptor 6; PLC, phospholipase C; PIP2, phosphatidylinositol 4,5-bisphosphate; IP3, inositol trisphosphate; DAG, diacylglycerol; PKC, protein kinase C. Fig. 2 was drawn using BioRender.

LTCCs are voltage-gated calcium channels (VGCCs) typically activated at membrane potentials between approximately –30 mV and –20 mV, with prolonged opening times that produce a stable calcium current [30, 31] (Fig. 2). Each LTCC consists of four subunits: α1, α2δ, β, and γ. The α1 subunit forms the core of the channel, facilitating ion conduction and imparting selective permeability to calcium ions [32]. Based on the genes encoding the α1 subunit, LTCCs are classified into four types: Cav1.1, Cav1.2, Cav1.3, and Cav1.4. Of these, Cav1.2 and Cav1.3 are particularly important in cardiac electrophysiological activity [33].

Cav1.2 is the predominant LTCCs subtype in cardiomyocytes, is widely distributed in atrial and ventricular cells, and plays a central role in myocardial excitation-contraction coupling (ECC) [34] (Table 1, Ref. [9, 33, 34, 35, 36, 37, 38, 39, 40, 41, 42, 43, 44, 45, 46, 47, 48, 49, 50, 51, 52, 53, 54, 55, 56, 57, 58, 59]). During the plateau phase (phase 2) of the action potential, Cav1.2 is activated, allowing a substantial influx of calcium ions into the cell. This influx triggers CICR via RyR2 on the SR, leading to muscle contraction [60, 61]. The synergistic interaction of Cav1.2 with other ion channels, such as voltage-gated sodium channels, IP3Rs, and TRP channels, further enhances calcium influx, supporting a rapid myocardial response and robust contraction [62, 63, 64].

**Table 1.** **Subtypes of calcium channels and their kinetics and pharmacology**.

Ca^2+^ channels		Activation potential	Modulators	Ref
Types	Subtypes			
LTCC channels	CaV1.1, CaV1.2, CaV1.3, CaV1.4	–50 to –20 mV, Slow inactivation	Verapamil, Diltiazem, Nifedipine	[33, 34, 35]
T-channels	CaV3.1, CaV3.2, CaV3.3	–70 to –40 mV, Rapid inactivation	Suvecaltamide, Nickel (Ni^2+^)	[36, 37, 38]
RyRs	RyR1, RyR2, RyR3	–30 mV to –20 mV	Ryanodine	[39, 40, 41]
IP3Rs	IP3R1, IP3R2, IP3R3	NA	IP3, Heparin, 2-APB	[42, 43, 44]
CRAC channels	Orai1, Orai2, Orai3, STIM1, STIM2	NA	2-APB	[45, 46, 47]
TRPC channels	TRPC1, TRPC3, TRPC4, TRPC5, TRPC6, TRPC7	–60 mV to +20 mV	SKF 96365	[9, 44, 48, 49, 50, 51, 52]
TRPM channels	TRPM1, TRPM2, TRPM3, TRPM4, TRPM5, TRPM6, TRPM7	–50 mV	9-Phenanthrol	[53, 54, 55, 56]
TRPV channels	TRPV1, TRPV2, TRPV3, TRPV4	–30 mV to –20 mV	Capsaicin	[57, 58]
TRPP channels	TRPP1, TRPP2	NA	Phenamil	[50, 59]

Notes: “NA” represents missing data. 2-APB, 2-Aminoethoxydiphenyl borate; T-channels, T-type calcium channel; CRAC, calcium release activated calcium; RyRs, ryanodine receptors.

Cav1.3 is primarily expressed in the sinoatrial (SA) and atrioventricular (AV) nodes, where it regulates the heart’s automatic pacing and conduction functions [35] (Table 1). Unlike Cav1.2, Cav1.3 has a lower activation voltage threshold, typically between –40 mV and –50 mV, enabling it to play a crucial role in the automatic depolarization of SA node cells and to trigger pacing activity at lower voltages [35, 63, 65]. In the AV node, Cav1.3 facilitates signal transmission between the atria and ventricles, coordinating atrial and ventricular contractions to maintain cardiac rhythm [66]. During repolarization, Cav1.3 contributes to signal transduction between the atria and ventricles. Additionally, LTCCs work in conjunction with small-conductance calcium-activated potassium channels (KCa2.x), facilitating the return of the cell membrane potential to its resting state by modulating potassium channel activity [67].

### 2.2 T-channels

T-channels are low-voltage-activated channels (LVACs), typically activated at membrane potentials near –60 mV (Fig. 2). They exhibit brief opening times, generating transient calcium currents and rapid inactivation [31]. The core structure of T-channels consists of the α1 subunit, which determines their selectivity, conductivity, and voltage dependence for calcium ions [68]. Based on the genes encoding the α1 subunit, T-channels are classified into three subtypes: Cav3.1, Cav3.2, and Cav3.3, encoded by the *CACNA1G*, *CACNA1H*, and *CACNA1I* genes, respectively [69].

Cav3.1 is primarily distributed in the SA nodes, AV nodes, and Purkinje fibers, where it contributes to cardiac pacing and rhythm control [36] (Table 1). Cav3.1 can be activated at low voltages near the resting membrane potential, resulting in a small calcium influx that triggers early depolarization. This activity aids the self-regulating cells of the SA nodes in reaching the threshold potential to initiate an action potential and subsequently activate Cav1.3 [70]. The expression level and functional strength of Cav3.1 determine the depolarization rate of the SA node, thereby modulating heart rate [71]. In the AV nodes and Purkinje fibers, Cav3.1 regulates calcium influx to delay the conduction of electrical signals, ensuring that atrial contraction is completed before ventricular contraction. This delay promotes synchronization between the atria and ventricles, which facilitates the coordination of cardiac function [37].

Cav3.2 is highly expressed in embryonic pacemaker cells and has been shown to contribute to rhythmogenic activity during early cardiac development [38]. However, in the adult heart, the role of Cav3.2 in normal pacemaker function remains controversial. While Cav3.2 expression is detectable in the sinoatrial and AV nodes, its contribution to adult pacemaker control appears to be limited. Genetic deletion studies indicate that Cav3.1, rather than Cav3.2, is the predominant T-type calcium channel responsible for ICaT (T-type calcium current) in adult rhythmogenic centers, as its knockout abolishes nearly all T-type calcium currents in these regions [72]. Nonetheless, Cav3.2 is more sensitive to acidic environments and oxidative stress, which may contribute to its role under pathological conditions such as AF [73]. Under stress conditions, Cav3.2 can activate delayed rectifier potassium channels, including HERG and Kir2.1, thereby accelerating cell membrane repolarization and potentially facilitating arrhythmogenic activity [74].

Although T-channels are not the primary pathway for calcium influx, they play a significant role in ectopic pacemaker activity and the highly excitable autonomic activity at the pulmonary vein ostium [75]. Studies have shown that upregulation of Cav3.1 and Cav3.2 increases the instability of atrial depolarization and enhances triggered activity, thereby promoting the occurrence of AF [76].

### 2.3 RyRs

RyRs are calcium release channels located on the membranes of the endoplasmic and SR, where they play a critical role in regulating intracellular calcium levels and contribute to calcium signaling and myocardial contraction [39] (Fig. 2, Table 1). RyRs are composed of four identical subunits that assemble into a large tetrameric complex, featuring three primary functional domains: the N-terminal regulatory domain, the central hub region, and the C-terminal pore domain. The N-terminal regulatory domain contains binding sites for calcium, calmodulin, and adenosine triphosphate (ATP), which modulate the channel’s opening. The central hub region relays signals to the C-terminal pore domain, thereby enabling calcium release [41].

In the heart, RyRs exist in three isoforms: RyR1, RyR2, and RyR3, with RyR2 being the predominant subtype in cardiomyocytes, and is extensively distributed on the SR membrane [40]. Upon depolarization of the cardiomyocyte membrane, Cav1.2 channels open, allowing calcium ions to enter the cytosol. This influx triggers the activation of RyR2 and initiates CICR, releasing large amounts of calcium from the SR to stimulate myocardial fiber contraction [41]. The localized calcium release from RyR2, known as “calcium sparks”, synchronizes across multiple sites to generate a calcium wave, leading to global myocardial contraction [77]. RyR2 also interacts with potassium channels, such as large-conductance calcium-activated potassium (BKCa) channels, to regulate membrane potential and modulate contraction strength via calcium sparks [78].

After contraction, calcium ions are reabsorbed into the SR by the SERCA, while the NCX extrudes a portion of calcium from the cell to maintain calcium homeostasis [79]. Calcium release from RyR2 is finely regulated through a dual mechanism: low cytosolic calcium concentrations activate RyR2, whereas elevated calcium concentrations inhibit its opening via a negative feedback mechanism, preventing calcium overload and cellular damage [80]. Additionally, calmodulin (CaM) binds to RyR2 in response to high cytosolic calcium levels, further inhibiting channel opening to protect against calcium overload [81].

Under conditions of sympathetic activation, such as stress or physical exercise, RyR2-mediated calcium release is enhanced through phosphorylation by protein kinase A (PKA) and calcium/calmodulin-dependent protein kinase II (CaMKII), leading to increased myocardial contractility [82]. However, abnormal activation of RyR2, particularly pathological calcium leakage, disrupts calcium homeostasis and destabilizes myocardial electrical activity. Studies indicate that RyR2-mediated calcium leakage can induce delayed afterdepolarizations (DADs), triggering ectopic excitation and thereby promoting the onset of AF [77]. This calcium leakage and subsequent calcium overload increase the heterogeneity of electrical activity in atrial myocytes, further exacerbating the maintenance and progression of AF [83].

### 2.4 IP3Rs

IP3Rs are key intracellular calcium release channels regulated by IP3 and reactive oxygen species (ROS) [84] (Fig. 2). IP3Rs are tetrameric protein complexes, each with a molecular weight of approximately 240–260 kDa, and are composed of three primary domains: the ligand-binding domain, the regulatory domain, and the pore domain [42]. Upon IP3 binding to the N-terminal ligand-binding domain, the calcium channel is activated. The regulatory domain modulates IP3R activity through interactions with calmodulin and phosphatidylinositol 4,5-bisphosphate (PIP2), while the C-terminal pore domain serves as the pathway for calcium efflux [85].

In mammals, IP3Rs exist in three isoforms: IP3R1, IP3R2, and IP3R3. IP3R2 is the predominant isoform in atrial myocytes, where it collaborates with RyR2 to regulate calcium dynamics within cardiomyocytes [42] (Table 1). In adult hearts, abnormal activation of IP3R2 is closely linked to pathological calcium overload, which can promote myocardial hypertrophy and arrhythmias [43]. Research indicates that IP3R2 plays a pivotal role in cardiac remodeling; its overactivation leads to intracellular calcium overload, exacerbating myocardial fibrosis and vascular contraction, thereby increasing the risk of AF and heart failure [86]. Recent studies suggest that targeting the structure of the mitochondria-associated endoplasmic reticulum (ER) membrane or modulating IP3R2 activity may effectively restore calcium homeostasis in cardiomyocytes, offering a potential therapeutic strategy to mitigate the progression of heart failure [87].

### 2.5 CRAC Channels

CRAC channels are non-voltage-dependent calcium channels (NVDCCs) primarily located in the pacemaker cells of the sinoatrial node and ventricular myocytes, where they are essential for maintaining cardiac calcium homeostasis [45] (Table 1). CRAC channels are composed of ORAI family proteins—ORAI1, ORAI2, and ORAI3—embedded in the cell membrane to facilitate extracellular calcium influx [47]. ORAI1 is the most highly expressed subtype in the heart, serving as the primary mediator of calcium signaling and homeostasis, while ORAI2 and ORAI3 function mainly in immune cells, providing complementary roles [45].

STIM1, a calcium-sensing protein located on the SR membrane, continuously monitors intracellular calcium levels [46] (Table 1). When calcium stores are depleted, STIM1 becomes activated and translocates to the cell membrane, where it binds to ORAI1 to form functional CRAC channels, enabling calcium influx through a process known as store-operated calcium entry (SOCE) [45] (Fig. 2). This influx of calcium directly affects cardiomyocyte depolarization, triggering additional calcium release and generating calcium transients essential for ECC in cardiomyocytes [88].

The cooperation between STIM1 and ORAI1, along with their interaction with VGCCs, enables precise regulation of calcium signaling. In conditions of calcium overload, CRAC channels work in concert with potassium channels to stabilize membrane potential [89]. However, excessive activation of CRAC channels in cardiomyocytes can lead to intracellular calcium overload, resulting in abnormal electrical activity and potential myocardial injury [47]. Therefore, modulating CRAC channel function may offer therapeutic benefits in maintaining calcium homeostasis and managing cardiac pathologies such as arrhythmias.

### 2.6 TRP Channels

The TRP channel family comprises various subtypes, including TRPC, TRPM, TRPV, and TRPP channels (Table 1). Notably, TRPC1, TRPC3, TRPC6, TRPM4, TRPM7, TRPV1, and TRPP2 are closely associated with cardiac function [9, 44, 48, 53, 54, 57, 90] (Fig. 2). Within the TRPC subfamily, TRPC1, TRPC3, and TRPC6 play key roles in cardiac contraction, myocardial hypertrophy, and arrhythmogenesis through the regulation of calcium influx. Abnormal activation of TRPC3 and TRPC6 has been linked to pathological cardiac remodeling [9, 44, 48]. TRPC3, in particular, modulates myocardial structure by influencing the proliferation and differentiation of cardiac fibroblasts, possibly via calcium influx in the extracellular signal-regulated kinase (ERK) signaling pathway [14]. Studies indicate that deletion of microRNA-26 enhances TRPC3 expression, further promoting the proliferation and differentiation of cardiac fibroblasts [91]. TRPC6 is also critical in cardiac fibroblast transformation, underscoring its role in myocardial remodeling [92].

Upon depletion of SR calcium stores, STIM1 protein activates and facilitates the interaction between ORAI2 and TRPC6, establishing a calcium signaling network that supports calcium homeostasis [93]. TRPC6 channels are activated by stimuli from G-protein-coupled receptors (GPCRs), mechanical stress, and fluctuations in sarcoplasmic calcium levels, thereby increasing calcium influx to sustain cardiomyocyte contraction and electrical activity [49]. TRPC6 activation elevates intracellular calcium concentrations, which promotes depolarization and the generation of action potentials, which contribute to pathological calcium dysfunction in the myocardium [9]. In addition to TRPC channels, TRPM4, a sodium channel, contributes to cardiomyocyte depolarization, thereby influencing cardiac automaticity and conduction [55]. TRPM7 is essential in maintaining calcium and magnesium homeostasis, and its dysregulation is linked to arrhythmias [56]. TRPM7 regulates HCN4 (hyperpolarization-activated cyclic nucleotide-gated channel 4) through epigenetic mechanisms and plays an important role in the control of heart rate, pacing and cardiac conduction [94]. HCN4 is the main pacemaker channel of SA cells, and its regulation by TRPM7 may directly affect the maintenance of heart rhythm [95]. TRPV1 is, responsive to temperature and chemical stimuli, regulates calcium influx, and affects cardiomyocyte excitability [58]. TRPP2 functions as a non-selective cation channel involved in calcium signaling, influencing cardiac structure and function [59]. The diversity of TRP channels and their regulatory roles in cardiac function make them an important area of interest. They are key to understanding the pathophysiology of cardiovascular diseases and identifying novel therapeutic targets.

## 3. Mechanisms of Calcium Channel Dysregulation in AF

When calcium channel function is impaired, calcium homeostasis is disrupted. Recent studies have shown that Cav1.2, Cav3.3, connexin 43, and RyR2 are highly expressed in the pulmonary veins of horses with AF, indicating a link between calcium channel dysregulation and AF [96]. Calcium homeostasis imbalance is recognized as a critical factor in the initiation and maintenance of AF. Increased calcium influx, abnormal calcium release, and impaired calcium transport mechanisms contribute to this imbalance [97]. This disruption in calcium homeostasis can lead to depolarization of the atrial myocyte membrane, enhancing atrial excitability and conductivity, thereby promoting the onset of AF [98]. Persistent calcium influx and calcium overload not only result in structural and functional remodeling of the atria—including atrial dilation, fibrosis, and impaired electrical conduction—but also facilitate the progression of AF from a paroxysmal to a persistent form, creating a vicious cycle that perpetuates AF [98, 99].

### 3.1 LTCCs Dysregulation

LTCCs are essential for maintaining calcium homeostasis in atrial myocytes. Under normal conditions, calcium ions enter the cell via LTCCs, triggering further calcium release from the SR to facilitate muscle contraction [60]. However, in AF, LTCCs dysfunction leads to either intracellular calcium overload or insufficient calcium influx, resulting in increased instability of calcium transients within atrial myocytes [41] (Table 2, Ref. [11, 12, 13, 14, 20, 31, 33, 41, 45, 49, 50, 51, 52, 75, 76, 79, 82, 98, 100, 101, 102, 103, 104, 105, 106, 107, 108, 109, 110, 111, 112, 113, 114, 115, 116, 117, 118, 119, 120, 121, 122, 123, 124, 125]).

**Table 2.** **Calcium channels dysregulation in AF**.

Ca^2+^ channels	Activation or inactivation	Ca^2+^ in cytoplasm	Electrophysiological impact	Ref
LTCC channels	↑	↑	Increased depolarization, prolonged action potential duration, leading to early afterdepolarizations (EADs) and triggered arrhythmias (TDP)	[11, 12, 31, 33, 100, 101]
	↓	↓	Decreased depolarization, shortened action potential duration, reduced myocardial contractility, leading to arrhythmias	[13, 41, 102, 103, 104, 105]
T-channels	↑	↑	Increased pacemaker activity, contributes to early depolarization, may enhance arrhythmogenicity	[20, 45, 75, 76, 98, 106, 107]
RyR2	↑	↑	Dysregulation of calcium release, leading to increased calcium spark events, contributing to arrhythmias	[82, 108, 109, 110, 111]
IP3Rs	↑	↑	Increased diastolic Ca^2+^ leak and arrhythmogenic potential	[112, 113]
CRAC channels	↑	↑	Enhanced calcium influx, contributing to cellular depolarization and arrhythmia initiation	[14, 114, 115, 116, 117, 118, 119]
TRPC channels	↑	↑	Increased calcium influx, contributing to pathological calcium overload and arrhythmias	[49, 51, 120, 121, 122, 123]
TRPM channels	↑	↑	Increased calcium influx, leading to cell membrane depolarization and arrhythmogenesis	[52]
TRPV channels	↑	↑	Increased calcium influx, contributing to cell excitability and arrhythmia	[123, 124]
TRPP channels	↑	↑	Increased calcium influx, leading to enhanced excitability and arrhythmogenic potential	[50]
NCX	↑	↓	Reduced calcium overload, leading to impaired contraction and potentially contributing to arrhythmia	[75, 79, 82, 125]

**Notes**: “↑” refers to the activation of calcium channels or changes in intracellular Ca^2+^ concentration. “↓” refers to the inactivation of calcium channels.

**Fig. 3.**
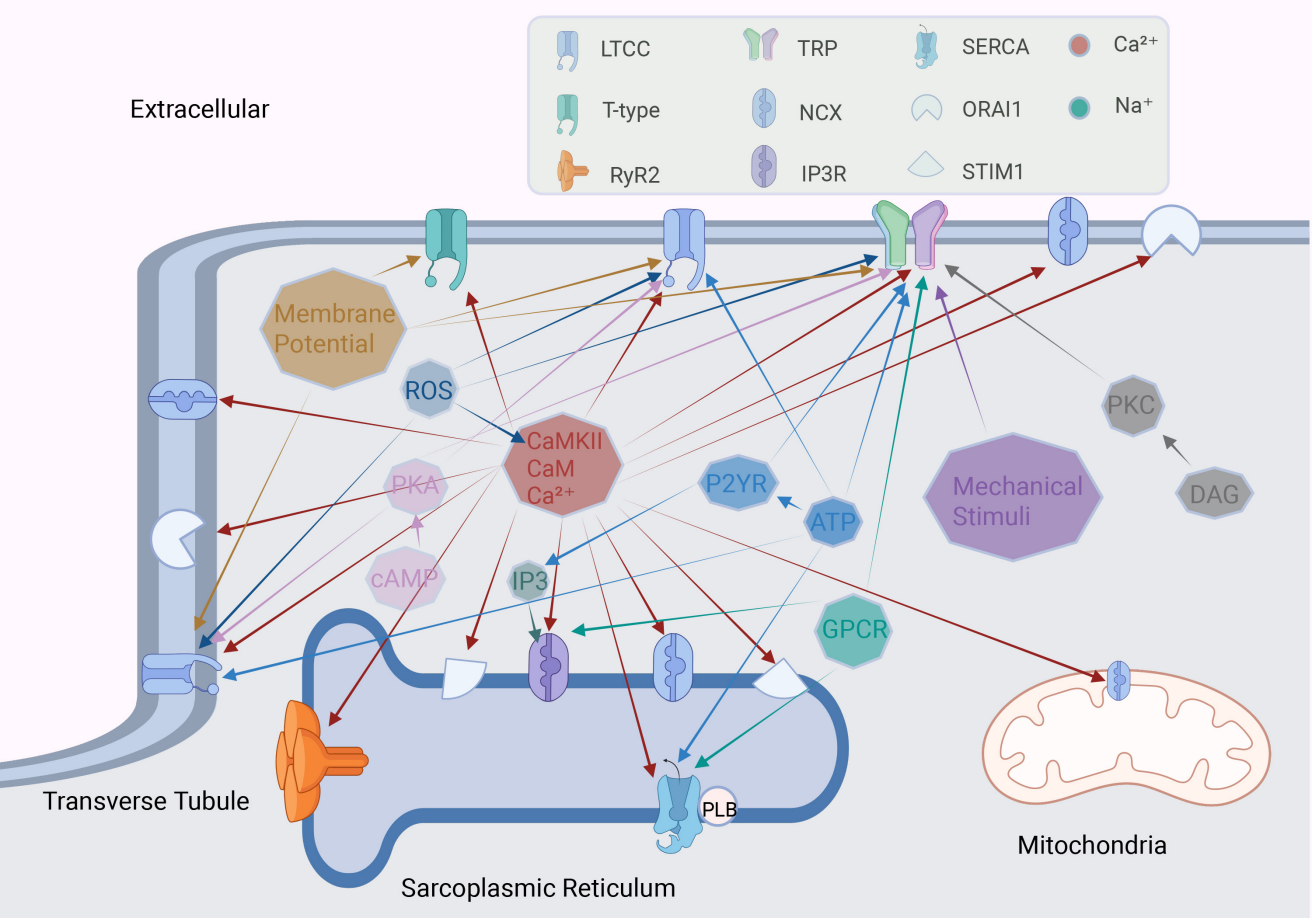
**Factors regulating calcium channels in cardiomyocytes**. The octagon represents different stimulating factors, and the arrow points to the calcium channel regulated by this factor. ROS, reactive oxygen species; PKA, protein kinase A; cAMP, cyclic adenosine monophosphate; CaMKII, calcium/calmodulin-dependent protein kinase II; CaM, calmodulin; P2YR, purinergic receptor P2Y; GPCR, G-protein coupled receptor. Fig. 3 was drawn using BioRender.

Upregulation of LTCCs enhances calcium influx and prolongs action potential duration (APD), thereby increasing the risk of reentrant arrhythmias [33]. Excessive calcium influx disrupts calcium transients, which can increase DADs, heightening the likelihood of triggered ectopic activity [11]. Additionally, calcium overload activates calcium-dependent non-selective cation channels (NSCCs), further aggravating atrial electrical instability through DAD generation [31]. AF is commonly associated with structural and electrophysiological remodeling of the atrial myocardium. The activation of CaMKII plays a key role in this process, promoting pathological changes such as atrial fibrosis and cellular hypertrophy [12] (Fig. 3). Research indicates that oxidative stress can further activate CaMKII, resulting in sustained activation of LTCCs, disrupting calcium homeostasis, and increased susceptibility to AF [100]. In addition, sympathetic nervous system activation upregulates LTCCs expression via β-adrenergic receptors and the cAMP/PKA signaling pathway, thereby enhancing calcium influx and exacerbating calcium imbalance [101].

In AF, the downregulation of LTCCs is primarily driven by decreased transcription and protein levels of the α1 subunit [102]. Experimental studies have demonstrated that intraperitoneal injection of lipopolysaccharide (LPS) significantly reduces LTCCs gene expression in SD rats [103]. *ETV1* gene knockout in mouse atrial myocytes has been shown to decrease Cav1.2 expression, shorten APD, and increase the incidence of recurrent excitations, potentially leading to structural remodeling such as myocardial fibrosis [13]. The electrical remodeling associated with AF also contributes to the down regulation of LTCCs, reducing the probability for channels to be opened, which leads to diminished calcium influx and decreased APD stability [102]. A decrease in calcium influx directly impacts Cav1.2 downregulation, weakening the CICR mechanism and disrupting intracellular calcium homeostasis. This impairment significantly reduces myocardial contractility, particularly in atrial myocytes, where the effects are more pronounced [41]. Furthermore, reduced calcium influx increases the burden on the SERCA, depleting calcium stores, and further aggravating calcium imbalance [27].

AF progression also modulates nuclear calcium levels in cardiomyocytes through the miR-26a-regulated IP3R1/CaMKII/HDAC4 signaling pathway, which accelerates LTCCs downregulation [104]. AF-induced nuclear translocation of NFAT downregulates Cav1.2 expression and reduces L-type calcium influx. This signaling cascade, coupled with miR-26 downregulation and subsequent enhancement of upstream potassium currents, promotes the persistence of AF [105].

### 3.2 T-channels Dysregulation

Upregulation of T-channels is closely associated with the initiation and maintenance of AF, along with molecular mechanisms involving CaMKII, oxidative stress, and other regulatory signaling factors [20] (Table 2, Fig. 3). CaMKII plays a dual role in this process: it phosphorylates and activates T-channels, while also enhancing the activity of RyR2. This dual activation exacerbates calcium leakage and overload, establishing a pathological feedback loop that perpetuates AF [106]. Ethanol exposure further contributes to AF susceptibility by upregulating T-channel expression through the PKC/GSK3β signaling pathway—a mechanism that is seen in alcohol-induced AF patients [107].

In AF patients, Cav3.1 upregulation increases intracellular calcium load in myocytes, promoting atrial electrical remodeling [76]. Similarly, Cav3.2 upregulation in response to myocardial ischemia and hypertrophy increases calcium influx, enhancing the automaticity of atrial myocytes and increases the risk of triggered activity [107]. The low-threshold activation characteristic of Cav3.1 allows it to initiate activity early in the action potential, contributing to early depolarizations and shortening the APD [70]. This shortening of the APD enhances atrial myocyte excitability, making reentrant electrical activity more likely and thereby sustaining AF [38].

Chronic AF results in further upregulation of T-channels, leading to increased abnormal calcium influx and significant alterations in the electrophysiological properties of atrial myocytes [98]. Excessive activation of T-channels results in calcium overload, which activates the NCX, triggering both early afterdepolarizations (EADs) and DADs, thereby facilitating the initiation and perpetuation of AF [75]. The resultant calcium overload from T-channel upregulation places an increased burden on the SERCA, impairing calcium reuptake and further disrupting calcium homeostasis. This dysregulation contributes to an imbalance in intracellular calcium levels within atrial myocytes, exacerbating AF pathology, and results in further disease progression [45].

### 3.3 RyR2 Dysregulation 

Calcium leakage through RyR2 channels is a central mechanism in the pathogenesis of AF. Under normal physiological conditions, RyR2 channels open in response to specific triggers, allowing tightly regulated calcium release from the SR [41] (Fig. 3). However, factors such as hyperphosphorylation and oxidative stress can lead to abnormal RyR2 activation, resulting in SR calcium depletion and sustained calcium leakage [108] (Table 2). Recent proteomic studies have identified that the absence of a novel regulatory subunit of protein phosphatase 1 (PP1), encoded by the PPP1R3A gene, accelerates the phosphorylation of RyR2 and phospholamban (PLN), increasing AF susceptibility in murine models [109]. Calcium leakage from RyR2 contributes to calcium overload within atrial myocytes, activating the NCX and inducing DADs during repolarization, which promotes ectopic activity and facilitates the initiation and maintenance of AF [79]. CaMKII and PKA are major pathways that contribute to RyR2 hyperphosphorylation, thereby exacerbating calcium leakage [82, 110, 111]. Elevated intracellular calcium levels further enhance CaMKII activity, which indirectly activates RyR2 by modulating LTCC and NCX function, creating a feedback loop that intensifies calcium dysregulation [82]. Studies have found that *ETV1* gene knockout in mice reduces Cav1.2 expression, affecting cardiomyocyte calcium homeostasis and RyR2 activation [13].

Clinical and experimental evidence has shown that RyR2 phosphorylation levels are significantly elevated in AF patients, leading to increased calcium leakage, particularly in the early stages of condition [109]. As AF progresses, this calcium leakage accelerates the transition from paroxysmal to persistent AF [126]. The chronic disruption of calcium homeostasis also drives atrial structural remodeling, including fibrosis and cardiomyocyte apoptosis, increasing atrial heterogeneity and thereby contributing to sustained AF pathology [15].

### 3.4 IP3Rs Dysregulation

Upregulation of IP3Rs has been linked to increased calcium influx, and increased susceptibility to AF (Table 2). Research indicates that combined activation of CaMKII and IP3 signaling pathways can stimulate IP3Rs, thereby promoting AF in patients with heart failure [112] (Fig. 3). ER stress and mitochondrial dysfunction are recognized as central mechanisms in atrial remodeling, especially in individuals with type 2 diabetes [127]. The IP3R1/GRP75/VDAC1 complex is crucial for calcium signaling between the ER and mitochondria, facilitating oxidative stress interactions that contribute to diabetes-associated atrial remodeling [128].

Excessive activation of IP3R2 has been shown to induce electrical remodeling in atrial myocytes, further increasing the risk of AF [113]. Anti-apoptotic members of the Bcl-2 family play a dual role: stabilizing mitochondrial membrane integrity and modulating IP3R activity within the ER, thereby directly influencing calcium dynamics and homeostasis [129]. These findings suggest that targeting IP3Rs and related regulatory pathways may offer a therapeutic approach to mitigate AF risk in patients with diabetes and heart failure [104, 130].

### 3.5 CRAC Channels Dysregulation

The CRAC channels, primarily consisting of STIM1 and ORAI1, play pivotal roles in the pathophysiology of AF, with dysregulated expression and function contributing significantly to the disease process [45] (Table 2, Fig. 3). In pathological states such as myocardial hypertrophy and heart failure, overactivation of ORAI1 leads to substantial intracellular calcium overload, thereby amplifying the calcium homeostasis imbalance. This disruption in calcium regulation results in aberrant electrical activity and myocardial fibrosis, key factors in the development and progression of AF [114]. SOCE, through enhanced collagen secretion by atrial fibroblasts, directly participates in the formation of atrial fibrosis, a process closely linked to the development of AF [115].

The interaction between CRAC channels and TRPC3 has also been demonstrated to stimulate the expression of fibrosis-related genes in atrial fibroblasts, further contributing to the persistence of AF [116]. In animal models, increased expression of ORAI1 and enhanced SOCE-mediated calcium influx have been strongly associated with the progression of atrial fibrosis, exacerbating the arrhythmic substrate [117, 118].

Clinically, elevated levels of ORAI1 expression in atrial tissues from AF patients have been found to correlate with increased calcium ion influx, triggering electrical remodeling and functional disturbances in the atria [14]. Recent research has highlighted fibroblast growth factor 23 (FGF-23) as a key regulator, which upregulates ORAI1-mediated calcium influx via activation of the FGF receptor 1. This discovery opens promising new avenues for targeted therapies in the management of AF [119].

### 3.6 TRP Channels Dysregulation

The role of TRP channels, particularly TRPC6, in the pathophysiology of heart disease, especially in the onset and maintenance of AF, has received significant attention in recent years. Studies have shown that the expression of TRP channel-related genes is elevated in leukocytes of patients with non-valvular AF (NVAF) [131]. TRPC1 activity may be triggered by angiotensin II or mechanical stress, leading to cardiomyocyte hypertrophy and fibrosis [120] (Table 2, Fig. 3). In conditions such as cardiac hypertrophy or arrhythmia, the synergistic effect of TRPC1 with CRAC channels enhances calcium influx, resulting in prolonged action potentials and spontaneous calcium release, thereby promoting electrical remodeling in the myocardium [49].

Upregulation of TRPC3, TRPC6, and TRPP2 has also been associated with myocardial remodeling, which contributes to cardiomyocyte hypertrophy and accelerates the progression of heart failure [50, 51]. In atrial tissue from AF patients, elevated TRPC6 expression is closely linked to the activation of NFAT and AP-1 transcription factors, which influence the electrophysiological properties of the heart by promoting TRPC6 gene expression [121]. Vericiguat has shown promise in AF management by attenuating structural and electrical remodeling through TRPC6 upregulation [132]. In heart failure models, TRPC6 upregulation has been observed to enhance contractility in cardiomyocytes, though it also leads to electrical instability [114]. Experimental models of AF have demonstrated that specific TRPC6 inhibitors can reduce the incidence of AF, underscoring the therapeutic potential of targeting TRPC6 [9, 133]. Other TRP family members also play significant roles in AF pathophysiology.

Mutations in TRPM4, a sodium channel, have been linked to idiopathic arrhythmias and cardiac conduction blockage [52]. TRPM7 deficiency or mutation disrupts calcium and magnesium homeostasis in cardiomyocytes and influences AF pathogenesis by modulating calcium and calmodulin signaling pathways [122, 134]. TRPV1, activated by oxidative stress and inflammation, is aberrantly expressed in atrial myocytes and may contribute to increased cellular stress responses, electrical remodeling, and persistence of AF [123, 124]. Overall, TRP channels represent critical therapeutic targets in AF pathophysiology. Modulation of TRP channel function offers a promising avenue for innovative treatments in arrhythmias and cardiomyopathies.

## 4. Application of Calcium Channel-Targeted Therapies in AF

Calcium channel-targeted therapies have attracted significant attention in the management of AF, particularly for their potential to stabilize atrial electrical activity by modulating calcium homeostasis.

LTCC blockers, including verapamil and diltiazem, are widely used in the management of chronic AF for effective heart rate control. These agents reduce calcium influx and prolong action potential duration in atrial myocytes, offering reliable rate control with a favorable safety profile [135]. Additionally, nifedipine significantly inhibits Cav1.2 by reducing connexin 43 levels, which may further contribute to its therapeutic effects [136]. Targeting the TGF-β signaling pathway and using agents such as sacubitril/valsartan or miR-155 have shown promise in downregulating Cav1.2 expression, thereby reducing AF-related fibrosis and electrical remodeling [137, 138]. Recent studies indicate that CRISPR-Cas9 gene-editing technology can selectively inhibit the *CACNA1C* gene, potentially correcting mutations associated with LTCC overexpression. This approach has demonstrated antifibrotic effects in human-induced pluripotent stem cell (iPSC) cardiomyocyte models, offering an innovative strategy for AF management pending further clinical validation [139, 140].

β-Blockers, which indirectly downregulate Cav1.2 by inhibiting sympathetic activity, are also used in AF management, although their atrial specificity is limited [141]. A small-molecule integrated stress response inhibitor has shown efficacy in reducing macrophage infiltration and oxidative stress following myocardial infarction in rat models, thus decreasing AF susceptibility [142]. Natriuretic peptide receptor B, which targets both LTCCs and RyR2, shows potential in treating AF triggered by β-adrenergic stimulation [143]. Additionally, KN-93, a CaMKII inhibitor, prevents LTCCs activation, making it a novel target for AF therapy [144].

Cav3.1 and Cav3.2 T-type calcium channels are essential in regulating cardiac automaticity. Studies have shown that TNF-α reduces T-type calcium channel currents in atrial myocytes of mice, while mibefradil suppresses Cav3.1 expression by downregulating connexin 43 [136, 145]. Additionally, low concentrations of isopimaric acid, a diterpene compound, have been found to inactivate both L-type and T-type calcium channels, leading to reduced action potential frequency and the restoration of normal cardiac rhythm [146]. Despite their potential, clinical applications of T-type calcium channel blockers are currently limited due to adverse effects. Consequently, the development of selective modulators for T-type calcium channels, with improved specificity and safety profiles, presents a promising direction for future AF therapies.

In AF patients, excessive phosphorylation of RyR2 leads to spontaneous calcium leakage, resulting in DADs and reentrant electrical activity. Modulation of RyR2 and the NCX via histone deacetylase inhibitors has shown promise in AF prevention [125]. Recent research has identified several agents—such as dapansutrile, febuxostat, the selective RyR2 inhibitor ent-verticilide, and propafenone, that can reduce AF susceptibility [147, 148, 149]. M201-A has demonstrated the potential to prolong the atrial effective refractory period and improve cardiac function without compromising ventricular contraction, making it a promising candidate for AF patients with concurrent heart failure [150].

In ischemic and cholinergic stimulation-induced models of AF, inhibition of the IP3R by 2-APB has been shown to effectively reduce cytosolic calcium overload and mitigate cellular energy damage [151]. Modulation of IP3R offers a promising approach not only to alleviate abnormal calcium signaling in AF but also to pave the way for the development of precision molecular therapies for arrhythmias. This targeted approach holds potential for advancing AF treatment through innovative molecular design and tailored therapeutic strategies.

The interaction between STIM1 and Orai1 is a crucial step in the activation of the CRAC channel. Inhibitors such as CM4620 and Synta66 have been shown to effectively block Orai1-mediated calcium influx, thereby preventing the trigger calcium transients that play a pivotal role in the initiation of AF [152, 153]. Oxidative stress is one of the key factors contributing to the abnormal activation of the CRAC channel, and its modulation may represent a potential therapeutic approach for AF [154, 155]. Qingyi decoction has been reported to regulate the STIM1/Orai1 pathway, mitigating inflammation and improving calcium homeostasis, which could provide a promising strategy for AF treatment [156]. Furthermore, enhancing the activity of the calcium ATPase SERCA2a accelerates calcium reuptake, which not only mitigates calcium overload but also helps restore calcium homeostasis, offering potential therapeutic avenues for managing AF-induced electrical remodeling [157].

In experimental models of diabetes, TRPC6 expression was significantly downregulated following treatment with Tinglu Yixin granules, leading to a reduction in inflammation, oxidative stress, and myocardial fibrosis [158]. In addition, studies using SKF96365 and puerarin have further demonstrated that the downregulation of TRPC6 expression can effectively mitigate myocardial remodeling and oxidative stress in diabetic rats [121, 159]. These findings suggest that targeting TRPC6 may hold therapeutic potential for alleviating the pathological changes associated with AF.

There are not many calcium channel therapeutic drugs in the clinical treatment of AF. At present, the most widely used calcium channel blockers are verapamil and diltiazem. Therefore, it is necessary to develop additional of calcium channel therapeutic drugs. Several emerging calcium channel regulation technologies are still in the laboratory stage, and the transformation from basic research to clinical application should be accelerated. Calcium channels are distributed in both the atrium and ventricle, and calcium channel modulators with high selectivity and low side effects will optimize AF treatment.

## 5. Conclusions

In the pathogenesis of AF, dysregulation of calcium channels disrupts calcium homeostasis, leading to electrophysiological dysregulation. and structural remodeling in the atrial myocytes. Various calcium channels—including L-type, T-type, RyRs, IP3Rs, CRAC and TRP channels—regulate intracellular calcium dynamics in unique and interrelated ways, driving the progression of AF.

While calcium channel-targeted therapies have shown efficacy in AF management, the diversity and complexity of calcium channel regulation in different atrial cell types continue to pose challenges for the development of highly specific treatments with minimal side effects. Additionally, strategies integrating multi-target drug design, gene editing, and precision medicine approaches to selectively correct calcium channel dysfunction may offer more promising therapeutic options for AF patients. These methods hold potential for optimizing AF management in the future, providing patients with more effective and personalized treatment options.

## Abbreviations

AF, atrial fibrillation; LTCCs, L-type calcium channels; T-channels, T-type calcium channels; RyRs, ryanodine receptor calcium release channels; IP3Rs, inositol 1,4,5-triphosphate receptors; CRAC channels, calcium release-activated calcium channels; TRP channels, transient receptor potential channels; STIM1, The binding of Stromal Interaction Molecule 1; SR, sarcoplasmic reticulum; CICR, calcium-induced calcium release; SERCA, sarcoplasmic/endoplasmic reticulum Ca^2+^-ATPase; NCX, sodium-calcium exchanger; ECC, excitation-contraction coupling; VGCCs, voltage-gated calcium channels; SA, sinoatrial nodes; AV, atrioventricular nodes; KCa2.x, small-conductance calcium-activated potassium channels; LVACs, low-voltage-activated channels; ICaT, T-type calcium current; BKCa, large-conductance calcium-activated potassium; CaM, calmodulin; PKA, protein kinase A; CaMKII, calcium/calmodulin-dependent protein kinase II; DADs, delayed afterdepolarizations; ROS, reactive oxygen species; PIP2, phosphatidylinositol 4,5-bisphosphate; NVDCCs, non-voltage-dependent calcium channels; SOCE, store-operated calcium entry; GPCRs, G-protein-coupled receptors; HCN4, hyperpolarization-activated cyclic nucleotide-gated channel 4; TDP, triggered arrhythmias; APD, action potential duration; NSCCs, non-selective cation channels; LPS, lipopolysaccharide; EADs, early afterdepolarizations; PP1, protein phosphatase 1; PLN, phospholamban; ER, endoplasmic reticulum; FGF-23, fibroblast growth factor 23; NVAF, non-valvular AF; iPSC, human-induced pluripotent stem cell.
